# Monitoring Autophagy in the Model Green Microalga *Chlamydomonas reinhardtii*

**DOI:** 10.3390/cells6040036

**Published:** 2017-10-22

**Authors:** María Esther Pérez-Pérez, Inmaculada Couso, Luis G. Heredia-Martínez, José L. Crespo

**Affiliations:** Instituto de Bioquímica Vegetal y Fotosíntesis, Consejo Superior de Investigaciones Científicas (CSIC)—Universidad de Sevilla; Avda. Américo Vespucio, 49, 41092 Sevilla, Spain; eperez@ibvf.csic.es (M.E.P.-P.); inmaculada.couso@ibvf.csic.es (I.C.); heredia@ibvf.csic.es (L.G.H.-M.)

**Keywords:** Chlamydomonas, green alga, autophagy, autophagic flux, ATG8, redox, stress

## Abstract

Autophagy is an intracellular catabolic system that delivers cytoplasmic constituents and organelles in the vacuole. This degradative process is mediated by a group of proteins coded by autophagy-related (*ATG*) genes that are widely conserved from yeasts to plants and mammals. Homologs of *ATG* genes have been also identified in algal genomes including the unicellular model green alga *Chlamydomonas reinhardtii*. The development of specific tools to monitor autophagy in Chlamydomonas has expanded our current knowledge about the regulation and function of this process in algae. Recent findings indicated that autophagy is regulated by redox signals and the TOR network in Chlamydomonas and revealed that this process may play in important role in the control of lipid metabolism and ribosomal protein turnover in this alga. Here, we will describe the different techniques and approaches that have been reported to study autophagy and autophagic flux in Chlamydomonas.

## 1. Introduction

**Figure 1:**
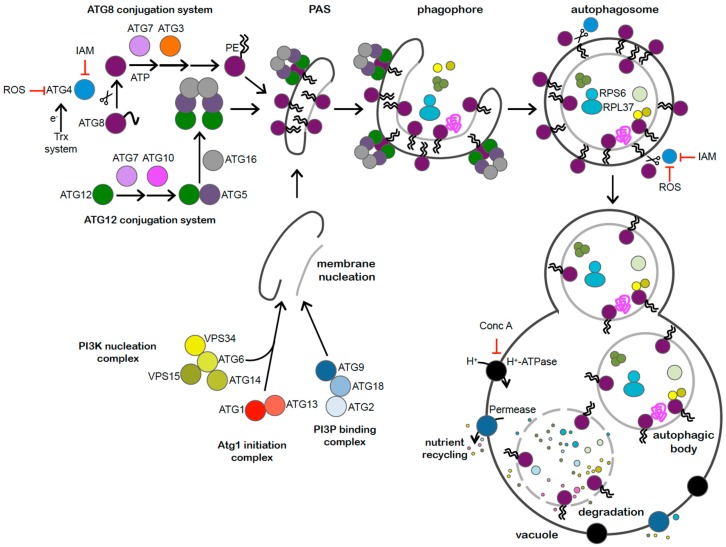
The autophagy machinery from Chlamydomonas. Most of the ATG proteins that compose the core autophagy machinery and participate in the formation of the autophagosome are conserved in Chlamydomonas. The ATG1 initiation complex, composed by ATG1 and ATG13, catalyzes the initial steps of autophagy. Then, the PI3K nucleation complex—constituted by ATG6, ATG14, VPS15 and VPS 34—participates in the membrane nucleation process and in the recruitment of PI3P binding complex formed by ATG2, ATG9, and ATG18, which is involved in the expansion of the phagophore. Next, the two conjugation systems, ATG8 ubiquitin-like system, composed by ATG8, ATG4, ATG7 and ATG3, and ATG12 ubiquitin-like system—composed by ATG12, ATG7, ATG10, ATG5, and ATG16—act coordinately to accomplish vesicle expansion, autophagosome formation, cargo recognition and autophagosome targeting to the vacuole. The ATG4 protease from Chlamydomonas is activated by the thioredoxin (Trx) system and reversibly inhibited by ROS or irreversibly inactivated by blocking agents such as iodoacetamide (IAM). Cytosolic components including ribosomal proteins such as RPS6 and RPL37 are engulfed by the autophagosome and targeted to the vacuole where they are finally degraded and recycled. Inhibition of vacuolar H+ ATPase with concanamycin A (Conc A) blocks autophagic flux. Accession numbers (from Phytozome https://phytozome.jgi.doe.gov/pz/portal.html) of Chlamydomonas ATG proteins are: ATG1 (Cre09.g391245.t1.1); ATG13 (Cre16.g659000.t1.1); ATG6 (Cre05.g242856.t1.1); VPS15 (Cre06.g290500.t1.1); VPS 34 (Cre01.g035500.t1.2); ATG2 (Cre01.g045600.t1.1); ATG9 (Cre09.g391500.t1.1); ATG18 (Cre10.g457550.t1.2); ATG8 (Cre16.g689650.t1.2); ATG4 (Cre12.g510100.t1.1), ATG7 (Cre03.g165215.t1.1) and ATG3 (Cre02.g102350.t1.2), ATG12 (Cre12.g557000.t1.2), ATG10 (Cre12.g532300), ATG5 (Cre14.g630907.t1.1) and ATG16 (Cre05.g242856.t1.1).

Eukaryotic cells are able to degrade intracellular material through a membrane-trafficking process known as autophagy. This catabolic process allows cells to deliver cytosolic contents including proteins, membranes, and even organelles to the vacuole or lysosome. The hallmark of autophagy is the formation of double membrane vesicles, termed autophagosomes, which engulf selectively or non-selectively cytosolic components. The autophagosome then fuses with the vacuole where the cargo is degraded by resident hydrolases (Figure 1). Autophagy usually occurs at a low basal level under optimal growth and is activated upon stress in order to maintain cellular homeostasis [1,2]. 

Autophagy is mediated by AuTophaGy-related (ATG) proteins, which were initially identified in yeasts [3], and subsequently in metazoans, plants, and algae [4,5,6,7]. Up to now, more than 40 ATG proteins have been described. Among them, the ubiquitin-like protein ATG8 plays an important role in the processes of autophagosome formation, target recognition, and vacuole tethering [8,9]. To develop its functions, ATG8 has to be anchored to the autophagosome membrane through the covalent binding to the membrane lipid phosphatidylethanolamine (PE). The lipidation of ATG8 to PE is catalyzed sequentially by the ATG8 conjugation system (Figure 1). First, ATG8 must be cleaved at a strictly conserved Gly at the C-terminus of the protein by the cysteine-protease ATG4. Then, by the consecutive action of the E1- and E2- activating enzymes, ATG7 and ATG3 respectively, and with the contribution of the ATG12–ATG5–ATG16 complex, ATG8 is finally bound to PE. Remarkably, ATG8 can be released from autophagosome outer membrane by the delipidating function of ATG4 [8], which may play an important role in the control of autophagy (Figure 1). The stable binding of ATG8 to the membrane has been widely used to monitor the process of autophagosome formation and a growing number of techniques are available to analyze ATG8 lipidation and autophagic flux in different organisms including algae [10]. Here, we discuss the principles, advantages, and limitations of the different approaches that have been used to study autophagy in the model unicellular green alga, *Chlamydomonas reinhardtii*.

## 2. Chlamydomonas as a Photosynthetic Model System to Study Autophagy

**Figure 2:**
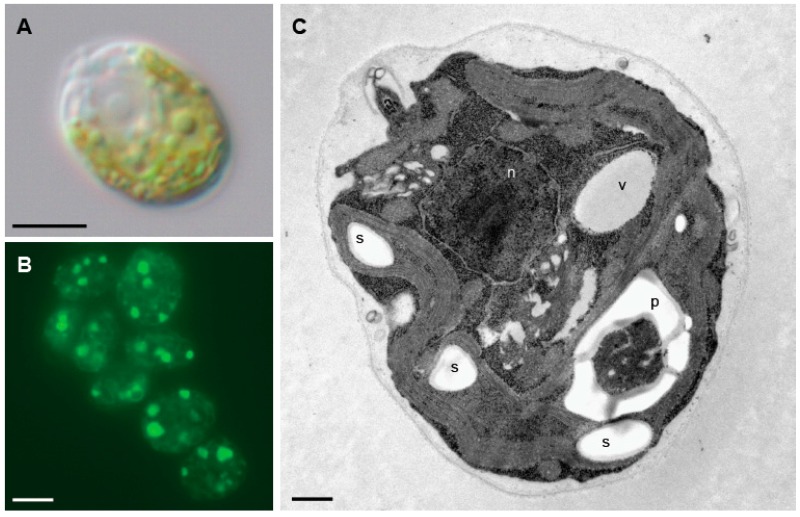
Microscopy images of Chlamydomonas cells. (**A**) Nomarski image of a Chlamydomonas cells. (**B**) Detection of lipid bodies by Nile red staining in Chlamydomonas cells under nitrogen limitation. (**C**) Ultrastructure of a Chlamydomonas cell. n, nucleus; p, pyrenoid; s, starch; v, vacuole. Scale bars: A and B, 5 µm; C, 500 nm.

The unicellular green alga, *Chlamydomonas reinhardtii* (referred to here as Chlamydomonas) is a well-developed model organism [11] that has been widely used for the study of important cellular and metabolic processes such as photosynthesis, cell cycle, redox regulation, flagella biogenesis, or nitrogen metabolism, among others [12,13,14]. Chlamydomonas cells are oval-shaped, possess two flagella at their anterior end and about 40% of their volume is occupied by a single cup-shaped chloroplast [15] (Figure 2). The Chlamydomonas genome was published about 10 years ago [16] and the most up-to-date genomic data is currently available at Phytozome (https://phytozome.jgi.doe.gov) after extensive assembly and annotation [17]. Chlamydomonas and other microalgae have recently emerged as valuable organisms since they are primary producers of natural and valuable products including vaccines, hydrogen, or nutraceuticals. Most algae produce oils, among them TAGs (triacylglycerols) are of special interest as they are considered a good source for biodiesel production [18]. Chlamydomonas is now considered as a model organism to study the TAG synthesis pathway since this alga can produce and accumulate high amounts of lipids in structures known as lipid bodies or lipid droplets under starvation conditions [19] (Figure 2). Chlamydomonas has also been proposed as a useful system for the study of autophagy in photosynthetic eukaryotes based on the easy handling of cell cultures for physiological and biochemical approaches and the reduced complexity of *ATG* genes in Chlamydomonas compared to higher plants [20,21]. Furthermore, Chlamydomonas displays a high metabolic plasticity since cells can grow in the presence (by means of photosynthesis) or absence of light (using acetate as carbon source), which provides unique physiological conditions among photosynthetic organisms to investigate the regulation of autophagy by light-derived stress signals. Accordingly, it has been shown that carotenoid depletion triggers autophagy in Chlamydomonas cells in the light but not in the dark [22].

Research on autophagy in Chlamydomonas is currently contributing to elucidating the regulation of this degradative process in photosynthetic organisms and has recently revealed an important role of autophagy in the control of lipid metabolism in algae. Inhibition of autophagy by the Target Of Rapamycin (TOR) kinase has been shown in algae since treatment of Chlamydomonas cells with the macrolide rapamycin results in increased vacuolization [23] and ATG8 lipidation [21]. None of these autophagy features were observed in an FKBP12 mutant strain treated with rapamycin [21,23], suggesting that a rapamycin-sensitive branch of the TOR signaling network inhibits autophagy in Chlamydomonas. Further work on the regulation of autophagy in Chlamydomonas revealed a strong link between the production of reactive oxygen species (ROS) and the activation of this process in photosynthetic organisms. Mounting evidence showed that autophagy is upregulated in Chlamydomonas in response to a wide range of stress conditions including nutrient limitation, oxidative stress, photo-oxidative damage, high light, endoplasmic reticulum stress, heavy metal toxicity, or salt stress among others [21,22,24,25,26,27,28]. The activation of autophagy in Chlamydomonas cells subjected to these stress conditions is linked to the generation of ROS and redox imbalance. Redox control of autophagy has been reported in other organisms including yeasts, mammals, and plants [29,30,31]. However, the molecular mechanisms underlying the redox regulation of autophagy are still poorly understood. So far, the ATG4 protease is the only ATG protein whose activity has been shown to be redox regulated. In humans, the activity of ATG4A/B is inhibited by oxidation in a process that involves a cysteine residue close to the catalytic cysteine [30]. The molecular mechanism for the redox regulation of ATG4 has been unraveled in yeasts and Chlamydomonas. It has been shown that ATG4 activity is similarly regulated in these two model systems by the formation of a single disulfide bond controlled by the thioredoxin system [29,32]. Furthermore, stress conditions that generate ROS and activate autophagy in Chlamydomonas promote the oxidation and aggregation of ATG4 in vivo. Specifically, carotenoid depletion induced by norflurazon or mutations in the phytoene synthase gene resulted in the activation of autophagy by photo-oxidative damage and the detection of ATG4 oligomers [32]. Thus, it has been proposed that the fine-tuning of ATG4 by the intracellular redox state may act as a regulatory hub for the redox control of autophagy [29,32]. Whether other ATG proteins are targeted by ROS remains unknown.

A recent study in Chlamydomonas revealed that inhibition of autophagic flux prevents the synthesis of TAGs and the formation of lipid bodies in nitrogen-limited cells [33]. Moreover, this study also showed that autophagic flux is needed for the recycling of some ribosomal proteins under nutrient stress conditions [33]. These findings strongly suggest that autophagy may play an important role in the regulation of lipid metabolism and ribosomal protein turnover in Chlamydomonas. Despite growing progress, autophagy is still poorly understood in Chlamydomonas mainly due to the lack of specific tools to monitor this catabolic process and the current limited knowledge on degradative vacuoles in this organism. How autophagy is initiated or which proteins catalyze the autophagosome-vacuole fusion needs to be investigated in Chlamydomonas. Nevertheless, a number of different approaches have been described to study autophagy in this model alga.

## 3. Methods for Monitoring Autophagy in Chlamydomonas

### 3.1. ATG8 Lipidation 

**Figure 3:**
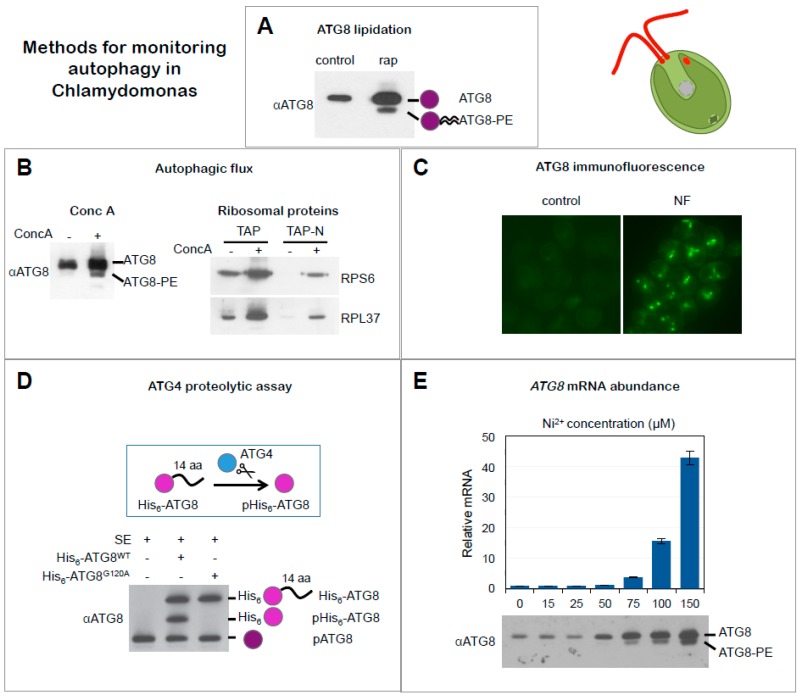
Methods for monitoring autophagy in Chlamydomonas. (**A**) ATG8 lipidation. Western blot analysis of ATG8 in cells growing exponentially in the absence (−) or presence (+) of rapamycin (rap) (adapted from [21]). (**B**) Inhibition of Autophagic flux by concanamycin A. Western blot analysis of ATG8 in cells treated (+) or not (−) with concanamycin A (ConcA) (upper panel). Monitoring autophagic flux by ribosomal protein degradation. Western blot analysis of RPS6 and RPL37 proteins from Chlamydomonas cells growing in rich medium (TAP: Tris acetate phosphate medium) or nitrogen-depleted medium (TAP-N) in the absence (−) or presence (+) of ConcA (lower panel) (adapted from [33]). (**C**) ATG8 localization. Immunolocalization of ATG8 in Chlamydomonas cells growing in exponential phase treated with norflurazon (NF). Untreated cells were used as control (adapted from [22]). (**D**) ATG4 proteolytic assay in cell-free extracts. Recombinant His_6_-tagged ATG8 protein (His_6_-ATG8^WT^) was incubated with Chlamydomonas cell-free soluble extracts (SE) and recombinant and endogenous ATG8 proteins were detected by Western blot. A glycine-to-alanine His_6_-tagged ATG8 mutant protein (His_6_-ATG8^G120A^) that cannot be processed by ATG4 was used as negative control. Unprocessed (His_6_-ATG8) and processed (pHis_6_-ATG8) forms of recombinant His_6_-ATG8 as well as endogenous ATG8 (pATG8) proteins are indicated (adapted from [21]). (**E**) ATG8 mRNA levels. Expression analysis of *ATG8* gene by quantitative RT-PCR (qPCR) in cells treated with increasing concentrations of nickel (Ni^2+^). Western blot analysis of ATG8 in the same conditions (adapted from [27]).

As described above, ATG8 stably associates with both inner and outer membranes of the autophagosome through covalent binding to PE in a process known as ATG8 conjugation or lipidation. Consequently, detection of lipidated ATG8 (ATG8-PE) has been widely used to monitor autophagy in multiple organisms [10]. The Chlamydomonas genome contains a single *ATG8* gene that encodes a ≈15 kDa protein with a C-terminal extension of 14 amino acids after the highly conserved Glycine residue [5,21]. A specific Chlamydomonas ATG8 antibody that recognizes free and lipidated ATG8 has been generated, and both isoforms can be clearly distinguished by SDS-PAGE followed by Western blot analysis since ATG8-PE migrates faster than the unmodified protein [21] (Figure 3A). The ATG8 antibody has been a valuable tool to monitor the lipidation state of this protein in Chlamydomonas. Under optimal growth, unmodified ATG8 is detected as a single band by Western blot that corresponds to the processed and unmodified form of the protein [21]. However, when Chlamydomonas cells are exposed to different stress conditions the abundance of this protein increases and lower apparent molecular mass corresponding to lipidated ATG8 can be also detected (Figure 3A). This approach has been successfully used in Chlamydomonas to investigate ATG8 lipidation under a broad range of stress conditions including nutrient limitation, TOR signaling inhibition, oxidative stress, photo-oxidative damage, or ER stress [21,22,28]. Moreover, a comparative analysis of ATG8 lipidation in wild-type and some mutant strains from Chlamydomonas revealed that cells defective in carotenoid biosynthesis [22] or chloroplast protease activity [34] display high levels of autophagy. Remarkably, the Chlamydomonas ATG8 antibody recognizes ATG8 proteins from other photosynthetic organisms such as the model plant *Arabidopsis thaliana* [21] and it has been used to monitor ATG8 lipidation in plants [35,36]. However, the high complexity of ATG8 proteins in plants (there are nine ATG8 isoforms in *A. thaliana*) hampers the unambiguous detection of lipidated forms of these proteins.

An important limitation when using ATG8 lipidation to monitor autophagy activity is that the accumulation of lipidated ATG8 does not necessarily reflect the activation of this process. For instance, we have recently reported in Chlamydomonas that inhibition of autophagic flux by concanamycin A, a specific inhibitor of vacuolar ATPase activity [37], results in an accumulation of ATG8-PE due to a blockage of vacuolar degradation and ATG8 recycling [33]. Nevertheless, an increased detection of ATG8 protein abundance and lipidation in response to stress usually indicates an upregulation of autophagy although this should be confirmed by other approaches such as immunolocalization of ATG8 or evaluation of autophagic flux.

### 3.2. Autophagic Flux

The autophagy process begins with the formation of the phagophore and ends with the degradation of the material sequestered by the autophagosome in the vacuole. The flow of material through the whole pathway is known as autophagic flux and it reflects the autophagic degradation activity within the cell [10]. A number of assays have been developed to determine autophagic flux in yeasts, mammals and plants by using a combination of techniques such as inhibition of vacuolar lytic function, Western blot analysis of specific key proteins, transmission electron microscopy and immunofluorescence microscopy [10]. These approaches have been recently defined for the study of autophagy in Chlamydomonas and provided valuable information about the role of this degradative process in algae [33].

The use of Chlamydomonas as a model system for the study of autophagy in algae has been hampered by the lack of *atg* mutants. The new released CLiP (Chlamydomonas Library Project) library, a collection of insertional mutants [38], might include some autophagy mutants, although no one has been confirmed yet (unpublished). In order to get around this problem, different approaches have been combined, firstly to inhibit the autophagy process and secondly to visualize it as described below. 

Concanamycin A as an inhibitor of autophagic flux: Detection of lipidated ATG8 by itself is an excellent marker for monitoring autophagy, but it only shows a snapshot of the process and needs to be combined with other methodologies. The analysis of ATG8-PE turnover combined with the use of concanamycin to block vacuolar degradation enabled visualization of autophagic flux in Chlamydomonas. It has been shown that both ATG8 and ATG8-PE accumulate in the absence of vacuolar degradation induced by concanamycin treatment, indicating the inhibition of the autophagy process (Figure 3B) [33]. A low concentration of concanamycin (0.1 µM) is sufficient to inhibit autophagy in Chlamydomonas [33]. In contrast, the use of other common inhibitors of autophagic flux like wortmannin [39] or 3-methyladenine [40] did not result in the detection of lipidated ATG8 in Chlamydomonas, which likely suggests that these drugs do not block autophagic flux in this alga [33]. Why wortmannin and 3-methyladenine have no effect on ATG8 lipidation in Chlamydomonas is currently unknown although it might be related to the low conservation of PI3K in this alga [33]. The use of concanamycin in Chlamydomonas combined with the detection of ATG8-PE is a convenient approach to monitor autophagic flux in this organism. This is in contrast to higher plants since detection of lipidated ATG8 forms following concanamycin treatment has not been a good marker for autophagic flux mainly due to the high complexity of ATG8 proteins in these organisms [41,42]. 

Ultrastructural analysis of Chlamydomonas cells treated with concanamycin by transmission electron microscopy revealed a higher degree of vacuolization and a pronounced increase of vacuole size. Moreover, large vacuoles could be observed in Chlamydomonas cells treated with concanamycin, suggesting that several vacuoles may merge to form a bigger one. At this stage, small vesicles have also been detected within the vacuoles of concanamycin-treated cells [33]. It has been reported that autophagic bodies accumulate in the vacuole of plant cells treated with concanamycin because vacuolar hydrolases cannot act [31,43,44], so the small vesicles detected inside the vacuoles of Chlamydomonas cells treated with concanamycin might also correspond to autophagic bodies.

Monitoring RPS6 and RPL37 as autophagic flux markers: Nitrogen starvation results in decreased abundance of ribosomal proteins [45] and activates autophagy in Chlamydomonas [21]. A recent study revealed that the downregulation of some ribosomal proteins is linked to the activation of autophagy in response to nitrogen or phosphate limitation [33]. The level of two ribosomal proteins, RPS6 and RPL37, largely decreased in nitrogen-starved cells whereas inhibition of autophagic flux by concanamycin prevented their degradation (Figure 3B) [33]. A similar result was obtained in Chlamydomonas cells upon phosphate limitation, which also triggers autophagy in this organism [33]. Based on these results, it has been proposed that RPS6 and RPL37 proteins might be part of the autophagosome cargo in nitrogen- or phosphate-starved cells and their abundance can be used to trace autophagic flux in Chlamydomonas. Whether the turnover of these ribosomal proteins takes place as part of a bulk degradation of cellular components or as a selective ribophagy process needs to be investigated. The downregulation of RPS6 and RPL37 in nitrogen- or phosphate-starved cells might be a good indicator of autophagic flux in Chlamydomonas, as described for NBR1 in plants [46,47], although it is currently unknown if the level of these proteins may also respond to other stress conditions.

### 3.3. Cellular Localization of ATG8

Cellular distribution of ATG8 by fluorescence microscopy has been analyzed in different organisms using fluorescent tags fused to the N terminus of the protein or specific antibodies raised against ATG8 [10]. In general, ATG8 proteins localize as small dots in the cytoplasm under optimal growth but in response to stress the number and size of spots significantly increase, reflecting the activation of autophagy and the formation of autophagosomes. Fluorescence microscopy studies have also been performed in Chlamydomonas to monitor the progress of autophagy under different stress conditions [21,22,27]. The cellular distribution of ATG8 in Chlamydomonas resembled the localization of this protein in yeasts. In rich medium, the ATG8 signal is usually weak and localized to discrete punctate structures. However, activation of autophagy by stress changed the localization of ATG8 in the cell and several spots with intense fluorescence are easily visible, in close agreement with the higher abundance of ATG8 protein detected by Western blot. So far, all stress conditions that trigger autophagy in Chlamydomonas result in a pronounced detection of ATG8 in punctate structures, indicating that ATG8 cellular distribution can be used to visualize the activation of autophagy in this alga (Figure 3C).

An important disadvantage of ATG8 immunolocalization assays by fluorescence microscopy in Chlamydomonas is the incompatibility of this approach for co-localization experiments with fluorescent-tagged proteins or dyes that require vital staining since cells need to be fixed and permeabilized for immunofluorescence detection. Therefore, it is technically challenging to co-localize ATG8 with other proteins unless monoclonal antibodies are available for the detection of these proteins. In this regard, the absence of reliable markers for different subcellular compartments in Chlamydomonas is a limiting factor when using the ATG8 antibody for cell biology studies in this alga. Nevertheless, a main advantage of immunofluorescence is that, unlike GFP-ATG8 fusion analysis, this approach allows the detection of endogenous ATG8, thus avoiding possible artifacts due to the overexpression of tagged proteins. Indeed, the Chlamydomonas ATG8 antibody has proven to be a convenient tool to analyze the cellular distribution of endogenous ATG8 in some mutant strains from Chlamydomonas [22,34].

### 3.4. ATG4 Proteolytic Assay

Unlike other ATG8 proteins, Chlamydomonas ATG8 has an extra amino acid sequence of 14 amino acids after the highly conserved glycine (Gly120) at the C-terminus that is recognized by the ATG4 protease (Figure 1 and Figure 3D). Therefore, full-length and mature ATG8 can be resolved by SDS-PAGE [21]. Taking advantage of the C-terminal extension of Chlamydomonas ATG8, the proteolytic activity of ATG4 can be analyzed in vitro using Chlamydomonas ATG8 as substrate. The C terminus of Chlamydomonas ATG8 is recognized and processed not only by Chlamydomonas ATG4 but also by yeast ATG4, which has been very useful to unravel the molecular mechanism underlying the redox regulation of ATG4 from Chlamydomonas and yeasts [29,32]. This assay was established initially to study the activity of purified ATG4 proteins in vitro, but a modified protocol has been also reported to determine the proteolytic activity of ATG4 present in total extracts from Chlamydomonas [21,32]. Briefly, a low amount of His_6_-tagged Chlamydomonas ATG8 is incubated with total extracts from Chlamydomonas cells and ATG4 activity present in the samples is analyzed by proteolysis of His_6_-ATG8 followed by SDS-PAGE and detection of ATG8 proteins by Western blot. The different size of endogenous ATG8 and the full-length and cleaved forms of recombinant His_6_-ATG8 allows the identification of the ATG4 proteolytic product, which can be measured for a quantitative analysis of ATG4 activity (Figure 3D). This assay has been used to demonstrate that ATG4 present in total extracts from Chlamydomonas is inactive in its oxidized form and can be activated by reduction [32]. A potential strength of this cleavage assay relies on its versatility since it might be extended to analyze the ATG4 protease activity present in total extracts from other organisms. However, the set-up of this approach is technically difficult and it may narrow its application to other organisms.

### 3.5. Transcriptional Activation of ATG Genes

As discussed above, activation of autophagy in Chlamydomonas results in a higher abundance of the ATG8 protein. It has also been shown that the induction of autophagy by different stresses correlates with an enhanced transcription of the *ATG8* gene in Chlamydomonas [27,28], strongly suggesting that the high level of the ATG8 protein is due to the upregulation of *ATG8* transcription. In response to stress, the autophagy machinery needs to be activated and this is in part accomplished by the transcriptional activation of some *ATG* genes that in turn leads to higher levels of the corresponding ATG proteins. In Chlamydomonas, it has been reported that ER stress, metal toxicity, oxidative stress, or rapamycin treatment activate the expression of the *ATG8* gene [27,28,34]. Moreover, global transcriptomic analysis of Chlamydomonas cells in which autophagy was induced by different stress signals revealed that other *ATG* genes such as *ATG3* and *ATG7* are upregulated in addition to *ATG8* [26,27,34,45]. Therefore, there seems to be a good correlation between the transcriptional activation of some *ATG* genes and the induction of autophagy in Chlamydomonas (Figure 3E). However, the quantitative analysis of *ATG* genes does not provide direct information about the activity of the autophagy process or its degradative capacity, and thus transcriptional studies should be complemented with additional approaches that specifically monitor autophagic flux such as detection of lipidated ATG8 in the presence and absence of vacuolar degradation, as recently shown in nitrogen- or phosphate-starved cells [33].

## 4. Perspectives

Autophagy research in algae is just taking off, but recent progress made on the regulation of this catabolic process and its possible link to the control of lipid metabolism in these photosynthetic organisms predicts a promising future to this new field. Growing lines of evidence revealed that autophagy is regulated by the intracellular redox potential in Chlamydomonas and that the ATG4 protease may integrate redox signals [21,22,32,48]. Moreover, it has been shown that the inhibition of autophagic flux in Chlamydomonas prevents the synthesis of TAGs and the formation of lipid bodies under nutrient limitation [33]. These results underscore the important role that autophagy may play in Chlamydomonas to maintain cellular homeostasis in response to stress. However, there is still much more to do to unravel the complex regulation of autophagy in photosynthetic organisms. A main disadvantage to studying autophagy in Chlamydomonas compared to other systems is the lack of specific tools. The availability of autophagy defective mutants from Chlamydomonas would certainly help to understand the function of this degradative process in algae, although the generation of stable and reliable knockout or knockdown mutants by RNAi approaches is still challenging in this organism. The absence of specific markers for some cellular compartments such as the vacuole, the ER or the Golgi is also a limiting step for co-localization studies with autophagy proteins. Efforts are currently focused on the generation of fluorescent tags fused to different ATG proteins in order to label and visualize the formation of autophagosomes in Chlamydomonas cells subjected to stress conditions in vivo. Together, these resources will enable a fast progress on our current understanding of the autophagy process and its regulation in Chlamydomonas that may have an impact on related systems such as higher plants.
